# Species recognition through wing interference patterns (WIPs) in *Achrysocharoides* Girault (Hymenoptera, Eulophidae) including two new species

**DOI:** 10.3897/zookeys.154.2158

**Published:** 2011-12-12

**Authors:** Ekaterina Shevtsova, Christer Hansson

**Affiliations:** 1 Department of Biology, Lund University, Sölvegatan 35, SE-22362 Lund, Sweden; 2 Scientific Associate of the Natural History Museum, Cromwell Road, South Kensington, London SW7 5BD, United Kingdom

**Keywords:** taxonomy, cryptic species, structural colours, sexual dimorphism, wing membrane thickness, Chalcidoidea, Entedoninae, leafminer parasitoids, *Achrysocharoides acerianus*, *Achrysocharoides platanoidae*, *Achrysocharoides robiniae*, *Achrysocharoides robinicolus*, *Achrysocharoides butus*, *Achrysocharoides latreilleii*, *Achrysocharoides albiscapus*, *Achrysocharoides maieri*, *Achrysocharoides serotinae*, *Phyllonorycter propinquinella*, *Phyllonorycter nr crataegella*, *Prunus serotina*, *Prunus pensylvanica*

## Abstract

Wing interference patterns (WIPs) are shown to be an important tool for species recognition in the genus *Achrysocharoides* Girault (Hymenoptera: Eulophidae). This is demonstrated by combining information from two previously published papers, comprising two cases of cryptic species, and by new material including the description of two new species, *Achrysocharoides maieri* and *Achrysocharoides serotinae* from North America. The cryptic species were initially separated through their distinct male WIPs. Subsequent analyses of the external morphology uncovered additional morphological differences supporting the original findings through WIPs, and biological data further strengthened the identity of these species. The new species described here also differ in their WIPs but the WIPs are similar in both sexes. Thus they provide a strong link between male and female and demonstrate that WIPs can also be useful for species recognition when the sexes are otherwise difficult to associate. Both new species are from Connecticut, USA, and were reared from *Phyllonorycter propinquinella* (Braun) (Lepidoptera: Gracillariidae) on black cherry (*Prunus serotina*); *Achrysocharoides maieri* has also been reared from *Ph. nr crataegella* on pin cherry (*Prunus pensylvanica*). To facilitate the identification of the new species they are included in a previously published key to North American species of *Achrysocharoides*. As a supplement to colourful WIPs we also demonstrate that grey scale images of uncoated wings from scanning electron microscopy can be used for visualization of the thickness distribution pattern in wing membranes.

## Introduction

**Figures 1–6.**
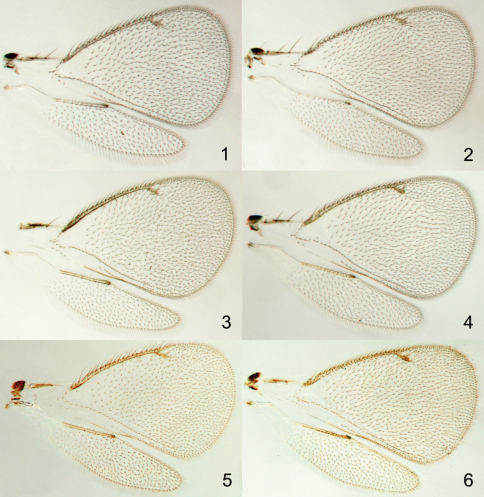
*Achrysocharoides* spp., transparent wings: **1** *Achrysocharoides acerianus* (Askew), male **2** Ditto, female **3** *Achrysocharoides platanoidae* Hansson & Shevtsova, male **4** Ditto, female **5** *Achrysocharoides butus* (Walker), male **6** Ditto, female. Wings on Figs **1–4** from Sweden, Skåne, 2010 **5–6** from Wales, 1976.

**Figures 7–9.**
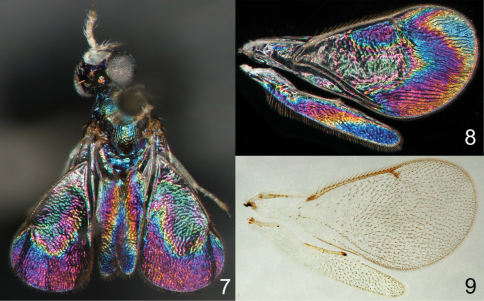
*Achrysocharoides* spp.: **7** *Achrysocharoides zwoelferi* (Delucchi), male, from Sweden, Blekinge, 1956 **8** Undescribed species from USA, Arizona, 1982, male, wing interference pattern (WIP) **9** The same wings as in Fig. **8** in transparent mode.

Species of *Achrysocharoides* Girault (Hymenoptera: Eulophidae) are small parasitic wasps with transparent non-pigmented wings (Figs 1–6, 9). The short postmarginal vein in the fore wing is characteristic for the genus and the shape of the fore wing can be used to distinguish males of some species, but otherwise wings have been disregarded as non-informative neutral entities in this genus (e.g. Askew and Ruse 1974; Kamijo 1991). Recently wings in this group were discovered to display patterns with stable structural colours (Fig. 7), comparable to other insect groups with colourful wings such as butterflies (Shevtsova et al. 2011). These wing interference patterns (WIPs) become visible when transparent insect wings are seen against a dark background, and are most distinctive in small species with exceptionally thin wing membranes.

WIPs as a morphological character are so new that very little is known about the significance of these patterns for their bearers or for entomologists studying them, although they have already proven useful for generic-level classification in Eulophidae (Hansson 2011). The application of WIPs as a species character was first used in a study including two cases of cryptic species in *Achrysocharoides* (Hansson and Shevtsova 2010), where the initial species separation was based solely on male WIPs. However, data showing the usefulness of WIPs were withheld pending the publication of Shevtsova et al. (2011) where a general background to these patterns was outlined. In order to expand the knowledge of WIP diversity and to prove the usefulness of these patterns for studies at the species level it is important to link the information from these two publications. To further enhance this knowledge we also describe two new *Achrysocharoides* species with distinct WIPs.

The two new species of *Achrysocharoides* described here are from North America and the genus was initially recorded from this region by Miller (1962), as the genus *Enaysma* Delucchi, including six new species from Canada which were placed in the same subgenus (*Pentenaysma* Graham). Yoshimoto (1977) synonymized *Enaysma* with *Achrysocharoides*, and added nine species (six newly described) to the six described by Miller. He also separated the 15 species into two newly created species groups, thus abandoning the division into subgenera. The latest comprehensive treatment of North American *Achrysocharoides* is by Kamijo (1991), who treated 18 species, including four new species and one new synonym, separated into five species groups, two of which were newly created. Hansson and Shevtsova (2010) added two new species to the North American fauna, increasing the total to 20 species. With the two new species described here this total is now 22, equal to the number of species in Europe. Worldwide, including the two new species described here, 56 species of *Achrysocharoides* are known. The majority (ten) of the remaining species are from Japan (Kamijo 1990a, b), thus establishing the main distribution of *Achrysocharoides* as the northern hemisphere.

## Material and methods

The observation and documentation of WIPs do not require a special light source and can be done on any dry specimen with intact wings arranged against a dark background. However, to make the illustrations comparable all photos in this paper as well as in Shevtsova et al. (2011) are of wings removed from the specimens and horizontally arranged, and with the same magnification (6×). To achieve this, the wings are flattened between a glass slide and a glass cover slip on top of the wings. The underside of the glass slide is stained with a drop of black ink to make the background pitch black and homogeneous (this was proposed by J. Kjærandsen). In a few cases where the wings could not be properly flattened the slide was slightly tilted so that the pattern in a non-flattened area, e.g. in a wrinkle, became visible and could be documented. This area was then manually combined in Adobe Photoshop with the initial horizontal photo of the wing, thus showing the complete pattern. A Nikon SMZ1000 stereomicroscope and 5MP Nikon DS-L1 camera were used to take photos of the wings at different focus levels, and Helicon Focus Pro version 4.75 software was used to merge them into a single image. WIPs are usually too shiny for the camera to balance brightness automatically and therefore the brightness was individually adjusted in Adobe Photoshop. Subsequent editing included cleaning and cropping of the photo. After the fore and hind wings were documented they were glued back to the card with the original specimen, which retained the second pair of wings for future observations – structural colours disappear on glued or slide mounted wings (Figs 1–6). The images of transparent wings in this paper are from temporary slide preparations with wings mounted in a water-soluble clear gel. The scanning electron microscopy (SEM) images (Figs 11, 13, 15, 17, 66–71, 82–87) are from uncoated specimens on their original card mountings. The photos were taken in low vacuum mode via a backscattered electron detector on a JEOL^®^ JSM 5600LV microscope.

**Figures 10–17.**
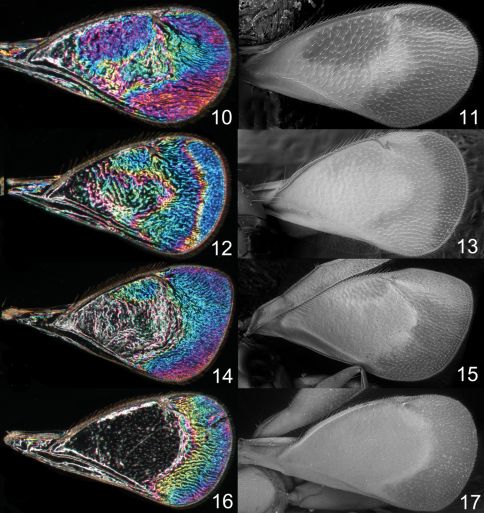
*Achrysocharoides* spp., males, wing interference patterns (WIPs) to the left, scanning electron micrographs from uncoated wings to the right: **10–11** *Achrysocharoides robiniae* Hansson & Shevtsova **12–13** *Achrysocharoides butus* (Walker) **14–15** *Achrysocharoides latreilleii* (Curtis) **16–17** *Achrysocharoides albiscapus* (Delucchi).

### Morphological abbreviations and acronyms

HE = height of eye; HW = height of fore wing; LG = length of gaster; LM = length of marginal vein; LW = length of fore wing, measured from base of marginal vein to apex of wing; MM = length of mesosoma; MS = malar space; OOL = distance between one posterior ocellus and eye; PM = length of postmarginal vein; POL = distance between posterior ocelli; POO = distance between posterior ocelli and occipital margin; ST = length of stigmal vein; WH = width of head; WM = width of mouth; WT = width of thorax. For illustrations of the morphological terms see http://www.neotropicaleulophidae.com/.

Collection acronyms, for the deposition of type material: BMNH = Natural History Museum, London, England; CAES = Connecticut Agricultural Experiment Station, New Haven, U.S.A; CNC = Canadian National Collection of Insects, Ottawa, Canada.

## Results and discussion

The paper by Hansson and Shevtsova (2010) included two cryptic *Achrysocharoides* species from *Acer*, *Achrysocharoides platanoidae* Hansson & Shevtsova from *Acer platanoides* and *Achrysocharoides acerianus* (Askew) from *Achrysocharoides pseudoplatanus*, and two cryptic speciesfrom *Robinia pseudoacacia*, *Achrysocharoides robiniae* and *Achrysocharoides robinicolus*, both described in that paper. The transparent wings in these four species are very similar and identical between males and females (Figs 1–4). Nevertheless the initial differences distinguishing these cryptic species were found in the wing morphology through distinct WIPs, which visualize uneven thickness of the wing membrane through different interference colours (Shevtsova et al. 2011).

**Figures 18–27.**
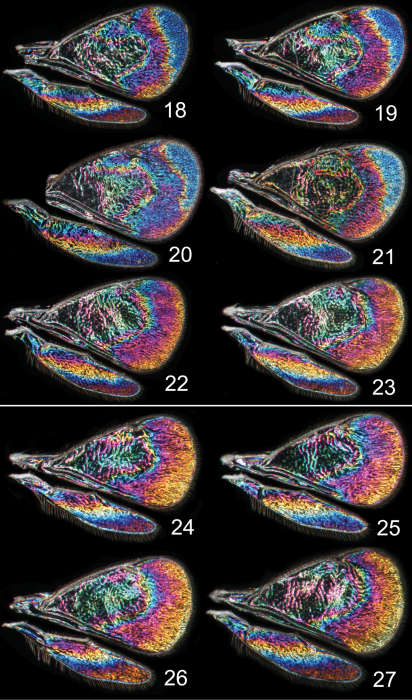
*Achrysocharoides* spp., wing interference patterns (WIPs): **18–23** *Achrysocharoides platanoidae* Hansson & Shevtsova **18–21** Males **22–23** Females **24–27** *Achrysocharoides acerianus* (Askew) **24–25** Males **26–27** Females. All wings from specimens from Sweden, Skåne, 2010.

**Figures 28–37.**
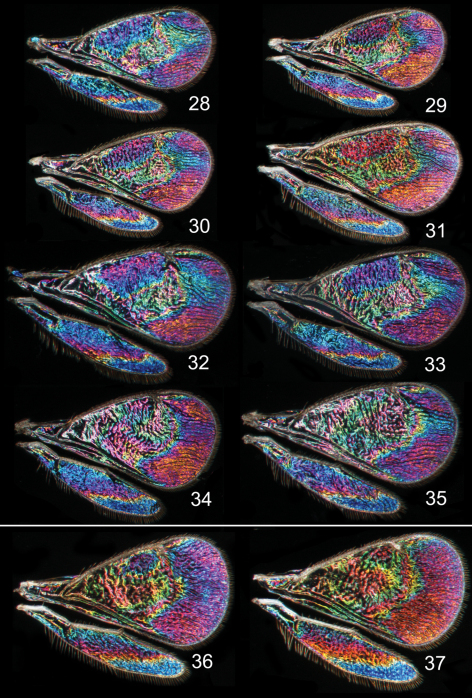
*Achrysocharoides* spp., wing interference patterns (WIPs): **28–35** *Achrysocharoides robiniae* Hansson & Shevtsova **28–33** Males **34–35** Females **36–37** *Achrysocharoides robinicolus* Hansson & Shevtsova **36** Male **37** Female. Wings on Figs **28–31, 34–37** from USA, Connecticut, 2002 **32, 33**, from Hungary, Vas Co., 2002.

In both cryptic cases only one of the species displays a distinct species specific WIP, and in males only, while conspecific females and both sexes of the other cryptic species have similar WIPs. In the two *Achrysocharoides* species associated with *Acer* only males of *Achrysocharoides platanoidae* have a distinctive WIP with an eye-catching blue spot in the upper-apical corner of the fore wing (Figs 18–21). The female WIP of *Achrysocharoides platanoidae* displays no such spot (Figs 22–23) and is very similar to *Achrysocharoides acerianus*, which has the same WIP in both sexes (Figs 24–27). In the two other cryptic species, associated with *Robinia pseudoacacia*, only males of *Achrysocharoides robiniae* display a very characteristic WIP with a large ovate spot below the marginal vein. The male WIP also has an extended and usually green triangular area in the medio-apical part of the fore wing (Figs 28–33). In the female WIP the triangular area is usually less pronounced than in males and the submarginal ovate spot is significantly smaller (Figs 34–35). As the female does not display the characteristic features in these patterns as distinctly as the male, it can be confused with the female of *Achrysocharoides robinicolus*, which has the same WIP in both sexes (Figs 36, 37).

The two North American species described here, *Achrysocharoides maieri* and *Achrysocharoides serotinae*, are known only from, and are probably confined to, *Phyllonorycter* species on *Prunus*. Males can be distinguished through easy-to-see differences in the external morphology, e.g. the shape of the head (Figs 57, 67, 73, 83) but females are not so distinct and display less divergent characters (Figs 56, 66, 72, 82). Even though the wings of *Achrysocharoides maieri* and *Achrysocharoides serotinae* appear very similar in transparent mode (similar to Figs 1–6) the WIPs in these species are distinct and specific. Apart from being useful in the discrimination of the females, in this case WIPs are also useful for the association of otherwise dimorphic males and females of the same species. The external morphology in these species exhibits a pronounced sexual dimorphism and as they share the same host it is not obvious which females and males are conspecific. However, there is one important character they have in common – WIPs, which are identical in both sexes but different between the species. *Achrysocharoides* *maieri* has a WIP with wide coloured cross bands on the fore wing (Figs 64–65), and *Achrysocharoides serotinae* has a quite featureless almost unicoloured WIP (Figs 80–81).

**Figures 38–45.**
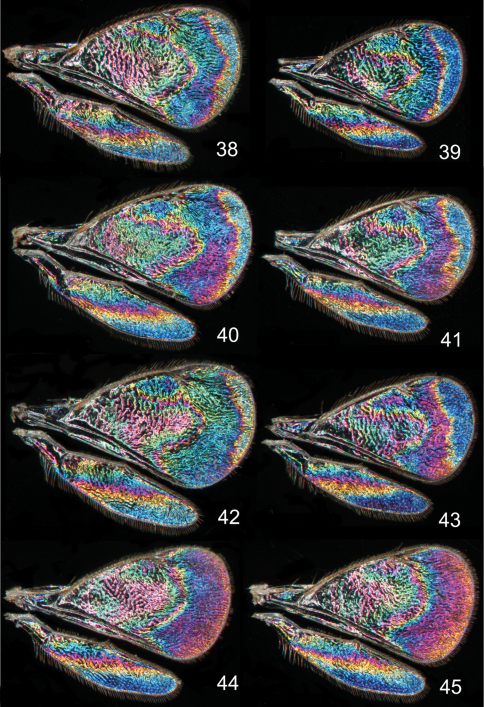
*Achrysocharoides* *butus* (Walker), wing interference patterns (WIPs): **38–43** Males **44–45** Females. Wings on Figs **38, 40–45** from Wales, 1976 **39** from Sweden, Skåne, 2010.

**Figures 46–55.**
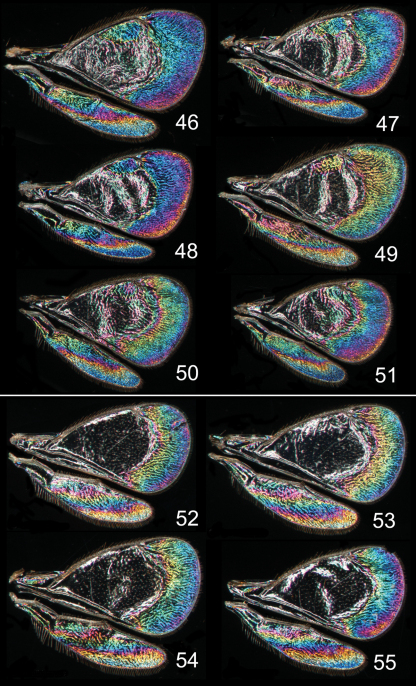
*Achrysocharoides* spp., wing interference patterns (WIPs): **46–51** *Achrysocharoides latreilleii* (Curtis) **46–49** Males **50–51** Females **52–55** *Achrysocharoides albiscapus* (Delucchi), males. Wings on Figs **46, 47, 49–51** from England, Surrey 1986–2004 **48** from Sweden, Skåne, 2010; **52, 53, 55** from Greece, Crete, 1997 **54** from France, 1984.

Additional examples of *Achrysocharoides* species with distinct and sexually dimorphic WIPs are *Achrysocharoides butus* (Walker) (Figs 38-43) and *Achrysocharoides latreilleii* (Curtis) (Figs 46-49) where characteristic and specific WIPs, again, are confined to males. Female WIPs of these two species are similar (Figs 44–45, 50–51), and as in females of *Achrysocharoides platanoidae* and *Achrysocharoides acerianus* (Figs 22–23, 26–27), and *Achrysocharoides robiniae* and *Achrysocharoides robinicolus* (Figs 34–35, 37), WIPs are not useful for species recognition. The WIP of male *Achrysocharoides butus* is similar to that of male *Achrysocharoides platanoidae* because the apical margin of the fore wing has a blue spot in both species. However, in *Achrysocharoides butus* this spot is prolonged and reaches along a major part of the apical margin (Figs 38–43) whereas in *Achrysocharoides platanoidae* the spot is short and confined to the upper-apical corner of the fore wing (Figs 18–21). The male of *Achrysocharoides latreilleii* is distinct not only in the truncate shape of the fore wing but also in its WIP (Figs 46–49). The basal 2/3 of the fore wing is the thickest part of the wing membrane and due to its micromorphology reflects very weak interference colours (Shevtsova et al. 2011). The apical part of the fore wing, and a small submarginal spot located in the corner between marginal and stigmal veins, are brightly coloured. The potential of WIPs as a character for separating species can be further demonstrated through two species where only male WIPs are known. *Achrysocharoides albiscapus* (Delucchi) has a WIP similar to that of *Achrysocharoides latreilleii*, but differs in having the basal 2/3 of the fore wing completely transparent without colour reflections and no submarginal colour spot (Figs 52–55). The shape of the fore wing is also different between males of these two species. The other species is undescribed, from Arizona, USA (specimen in CNC), and we have only seen a single male. This specimen has a distinctive WIP which emphasizes very unusual shapes of both fore and hind wings. The WIP includes a blue spot in the upper-apical corner of the fore wing (Fig. 8), comparable to *Achrysocharoides platanoidae* (Figs 18–21) but with the blue spot smaller and differently shaped.

Similar to other morphological characters there is a certain intraspecific variation in WIPs (Figs 18–55), but the species specific traits nevertheless remain clearly recognizable and are reliable for species separation. The intraspecific variation in WIPs can be divided into two types, variation in colour and in shape of patterns. Variation in colour is basically size-dependent – the thickness of the wing membrane usually varies with the size of the specimen and there is a general shift of the hues in WIPs from larger to smaller specimens. Variation in the shapes of pattern outlines of conspecific WIPs is not apparently size dependent but reflects individual differences between specimens - the overall pattern nevertheless remains the same.

Wing interference patterns are due to structural organization patterns of the wing membrane where areas of different thickness reflect certain interference colours (Shevtsova et al. 2011). We have found that the uneven thickness of the wing membrane also can be demonstrated and authenticated through the contrast in grey scale SEM images of uncoated wings. The SEM images created through back-scattered electrons (BSEs) visualize specific patterns on wings (Figs 11, 13, 15, 17). These patterns fully correspond to the approximate mapping of the wing thickness based on WIPs where the thickness of the wing membrane at any point can be estimated by the reflected interference colour (Shevtsova et al. 2011). The thickness gradient as seen through grey scale gradients in SEM images is due to specific properties of BSEs which have the escape depth of up to hundreds of nanometers (Egerton 2005). This means that the signal comes from a sample depth in the range comparable to membrane thickness in wings producing bright WIPs, i.e. 100–600 nm. In uncoated wings the primary (incident) electrons are scattered inside the membrane and reflected as BSEs to the back-scatter detector. In thick areas of the membrane the amount of BSEs is large, resulting in a strong signal, while thin areas of the membrane produce fewer BSEs and a weaker signal, thus displaying light and dark grey hues respectively. If the wings are coated with platinum or gold, the resulting picture is completely different due to secondary electrons (SEs) which are generated only within a very small distance below the surface as the escape depth of SEs is less than two nanometers (Egerton 2005), thus displaying the surface of the specimen rather than the underlying structure. In Shevtsova *et* *al*. (2011) secondary electron images were used to illustrate the microstructures of the wing surface, such as the ridges of membrane corrugations with rows of setae.

The clarification of the two cryptic species on *Acer* spp. (Hansson and Shevtsova 2010, Shevtsova et al. 2011) requires a correction of the molecular information deposited in Genbank. At the time of the publication of Lopez-Vaamonde et al. (2005) the identity of the *Achrysocharoides* species associated with *Acer* spp. was not clear, and “Achrysocharoides acerianus ex Acer platanoides” and “Achrysocharoides sp. ex Acer pseudoplatanus” in Lopez-Vaamonde et al. (2005) are *Achrysocharoides platanoidae* and *Achrysocharoides acerianus* respectively, which is confirmed here with new molecular analyses compared to data of “Achrysocharoides sp.” and “A. acerianus” in Genbank. Our new sequences include CO1, 18S, 28S and will be deposited in Genbank.

## Species descriptions

### Achrysocharoides maieri sp. n.

urn:lsid:zoobank.org:act:2E20B2E3-557F-413E-8729-D9ADD646FE3C

http://species-id.net/wiki/Achrysocharoides_maieri

Figures 56 –71

#### Material.

HOLOTYPE male (CNC) glued to a card, labeled “U.S.A.: Connecticut, New Haven Co., New Hamden, Lockwood Farm, 1.viii.1980, C.T. Maier”, “Tentiform mine of Phyllonorycter propinquinella on Prunus serotina, emerged in laboratory within 3 weeks”. PARATYPES: 1 female 3 males with same label data as holotype (BMNH, CAES, CNC)); 1 male labeled “U.S.A.: Connecticut, Tolland Co., Willington, 21.x.1981, Chris T. Maier”, “Mines of Phyllonorycter propinquinella on black cherry, Prunus serotina, on 21.x.1981, chilled outdoors, parasitoid emerged in laboratory in April 1982” (CNC); 3 females “U.S.A.: Connecticut, New Haven Co., North Haven, 1.vii.1981, C.T. Maier”, “Tentiform mine of Phyllonorycter nr crataegella on Prunus pensylvanica, emerged in laboratory within 3 weeks” (BMNH, CNC); 2 females 1 male from same locality and same host as previous but collected 2.vi.1986 (BMNH).

#### Diagnosis.

Both sexes: fore wing WIP with several distinct wide colourful cross-bands traversing the wing (Figs 64, 65), fore coxa white, hind coxa except apex golden green (Figs 62, 63); male: scape widest just below median part, with a single sparse row of setae along ventral margin (Fig. 59), antennal scrobes join frontal suture wide apart (Figs 67), vertex with long forward pointing setae (Fig. 69) – setae about as long as distance between posterior ocelli, upper frons without setae (Fig. 67), frons very large and wide (Fig. 67) - at its widest part 0.8X as wide as width of head; female: scape predominantly white and widest medially, with a single row of setae along ventral margin, propodeum smooth (Fig. 70), frons above frontal suture with raised and strong reticulation (Fig. 66).

#### Description.

**Figures 56–65.**
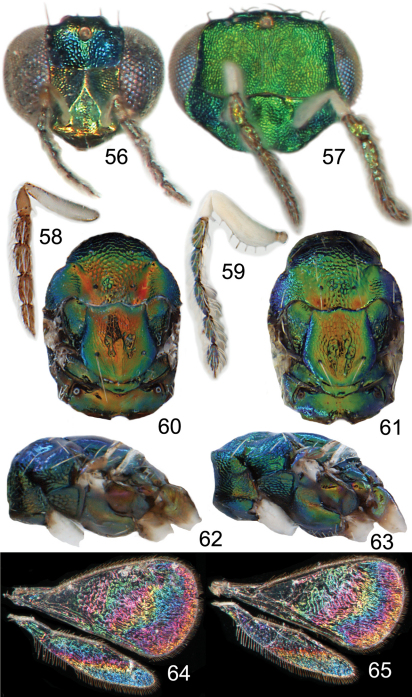
*Achrysocharoides* *maieri* sp. nov.: **56** Head frontal, female **57** Ditto, male **58** Antenna lateral, female **59** Ditto, male **60** Mesosoma dorsal, female **61** Ditto, male **62** Mesosoma lateral, female **63** Ditto, male **64** Wing interference pattern (WIP), female **65** Ditto, male.

*Female*. Length 1.1–1.5 mm. Scape white with inner apical tip infuscate; pedicel pale brown; flagellum dark brown (Fig. 58). Frons below frontal suture golden green to golden red, above frontal suture bluish green metallic (Fig. 56). Vertex inside ocellar triangle golden red, outside ocellar triangle golden green. Mesoscutum golden green with golden red areas – especially so in smooth posterior notaular depressions, to completely golden green (Fig. 60). Scutellum golden red with sides and posterior margin bluish green metallic, to completely golden green (Fig. 60). Propodeum golden red to golden green (Fig. 60). Fore coxa white, mid coxa dark brown with apical 1/3 white to completely dark brown, hind coxa golden green (Fig. 62); femora, tibiae and tarsi on all legs white. Wings without pigmented areas; WIP in fore wing with several distinct wide colourful cross-bands traversing the wing (Fig. 64). Gaster with first two tergites golden green, remaining tergites golden purple with green metallic tinges.

**Figures 66–71.**
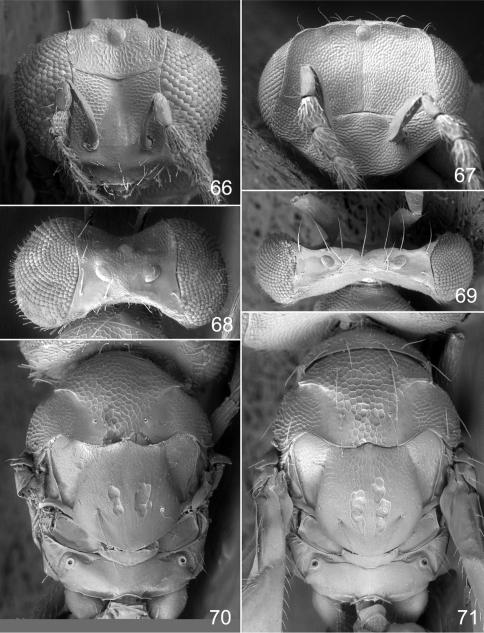
*Achrysocharoides* *maieri* sp. n.: **66** Head frontal, female **67** Ditto, male **68** Vertex, female **69** Ditto, male **70** Mesosoma dorsal, female. **71** Ditto, male.

Antenna as in Fig. 58. Frons below level of toruli smooth and shiny (Fig. 66), between level of toruli and frontal suture with raised and strong reticulation lateral to antennal scrobes, between antennal scrobes with very weak reticulation, above frontal suture with raised and strong reticulation. Vertex inside ocellar triangle with engraved and weak reticulation, outside ocellar triangle smooth and shiny (Fig. 68). Occipital margin rounded.

Pronotal collar without transverse carina (Fig. 70). Mesoscutum with raised and strong reticulation (Fig. 70), meshes of reticulation smaller on sidelobes than on midlobe, midlobe with pits (i.e. with very strong reticulation) posteromedially; notauli as smooth impressions in posterior 2/3. Scutellum with very weak reticulation and shiny, smooth along posterior margin, with 3–4 pits medially on either side of imaginary median longitudinal line (Fig. 70). Dorsellum flat and smooth, anterolaterally with two foveae. Propodeum smooth and shiny (Fig. 70); propodeal callus with three setae. Fore wing speculum closed below. Petiole conical without shoulders.

**Ratios**. HE/MS/WM = 5.0/1.0/2.3; POL/OOL/POO = 2.6/1.1/1.0; WH/WT = 1.2; LW/LM/HW = 1.6/1.0/1.0; PM/ST = 1.0; MM/LG = 0.8–0.9.

*Male*. Length 1.4–1.5 mm. Scape and pedicel white; flagellum dark brown with golden green tinges (Fig. 59). Frons green metallic (Fig. 57). Vertex inside ocellar triangle golden red, outside ocellar triangle golden green. Mesoscutum golden green with posterior 1/3 of notaular depressions golden red (Fig. 61). Scutellum golden red with sides bluish green metallic (Fig. 61). Propodeum golden green (Fig. 61). Fore coxa white, mid coxa dark brown with apical 1/3 white to completely dark brown, hind coxa golden green with apical half white (Fig. 63); femora, tibiae and tarsi on all legs white. Wings without pigmented areas; WIP very similar to that of the female (Fig. 65). Gaster with tergites 1–2 golden green with a large white spot medially, remaining tergites dark brown with purple metallic tinges.

Antenna as in Fig. 59, i.e. scape widest just below middle. Frons with engraved and strong reticulation (Fig. 67); antennal scrobes reaching frontal suture wide apart; transverse ridge straight medially. Vertex inside ocellar triangle with engraved and very weak reticulation, outside ocellar triangle smooth and shiny (Fig. 69); anterior part with a row of seven long and proclinate setae. Occipital margin rounded.

Pronotal collar without transverse carina (Fig. 71). Mesoscutum with raised and strong reticulation (Fig. 71), meshes of reticulation smaller on sidelobes than on midlobe, midlobe with pits (i.e. with very strong reticulation) posteromedially; notauli as smooth impressions in posterior 2/3. Scutellum very weak reticulation and shiny, smooth along posterior margin, with 3–4 pits medially on either side of imaginary median longitudinal line (Fig. 71). Dorsellum flat and smooth, anterolaterally with two foveae. Propodeum smooth and shiny (Fig. 71); propodeal callus with three setae. Fore wing speculum closed below. Petiole conical without shoulders.

**Ratios**. HE/MS/WM = 2.3/1.0/1.3; POL/OOL/POO = 14.4/6.4/1.0; WH/WT = 1.4; LW/LM/HW = 1.5/1.0/1.0; PM/ST = 1.0; MM/LG = 1.0.

#### Etymology.

Named after Dr. Chris T. Maier, Entomologist at the Connecticut Agricultural Experiment Station, who collected all material of the two new species described here.

#### Distribution.

U.S.A. (Connecticut).

#### Hosts.

*Phyllonorycter propinquinella* (Braun) (Lepidoptera: Gracillariidae) on black cherry (*Prunus serotina*), and *Phyllonorycter nr crataegella* on pin cherry (*Prunus pensylvanica*).

### Achrysocharoides serotinae sp. n.

urn:lsid:zoobank.org:act:C0EA95FF-793E-46BF-AF38-E300F345AB48

http://species-id.net/wiki/Achrysocharoides_serotinae

Figures 72 –87

#### Material.

HOLOTYPE male (CNC) glued to a card, labelled “U.S.A.: Connecticut, New Haven Co., North Haven, 30.ix.1981, Chris T. Maier”, “Adult parasitoid lab-reared from tentiform mine of *Phyllonorycter propinquinella* collected on black cherry, *Prunus serotina* on 30.ix.1981”. PARATYPES: 1 male with same label data as holotype (CNC); 2 females labeled “U.S.A.: Connecticut, Tolland Co., Union, 23.vi.1981, Chris T. Maier”, “Adult parasitoid lab-reared from tentiform mine of *Phyllonorycter propinquinella* collected on black cherry, *Prunus serotina* on 23.vi.1981” (CNC).

#### Diagnosis.

Both sexes: fore wing WIP almost unicoloured, gradually changing hue from purple to green towards the margin, without any distinct details such as lines or spots (Figs 80, 81), fore coxa predominantly dark brown, hind coxa golden green (Figs 78, 79); male: scape with about same width throughout, with a single sparse row of setae along ventral margin, antennal scrobes joining on frontal suture (Fig. 73, 83), vertex with long forward pointing setae (Fig. 85) – setae at most as long as distance between posterior ocelli, upper frons without setae (Fig. 83); female: scape pale brown and widest medially, with a single row of setae along ventral margin (Fig. 74), propodeum smooth (Fig. 86), frons above frontal suture with raised and strong reticulation (Fig. 82).

#### Description.

**Figures 72–81.**
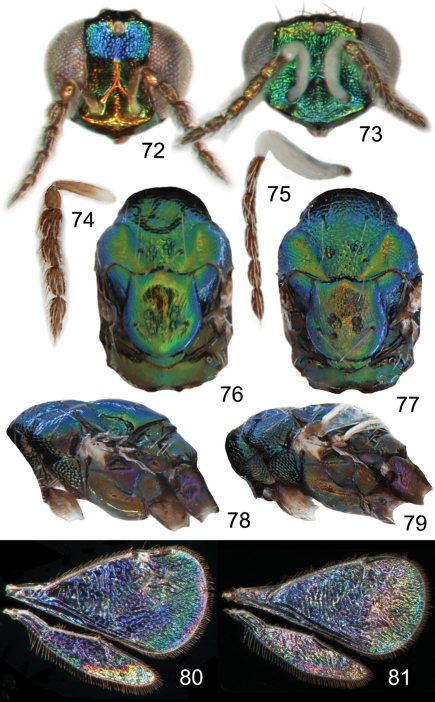
*Achrysocharoides* *serotinae* sp. n.: **72** Head frontal, female **73** Ditto, male **74** Antenna lateral, female **75** Ditto, male **76** Mesosoma dorsal, female **77** Ditto, male **78** Mesosoma lateral, female **79** Ditto, male **80** Wing interference pattern (WIP), female **81** Ditto, male.

*Female*. Length 1.2–1.3 mm. Scape and pedicel pale brown; flagellum dark brown (Fig. 74). Frons below frontal suture golden red, above frontal suture bluish green metallic (Fig. 72). Vertex inside ocellar triangle golden red, outside ocellar triangle golden green. Mesoscutum green metallic with blue metallic tinges, smooth parts of notaular depression golden green (Fig. 74). Scutellum golden green with sides and posterior margin bluish green metallic (Fig. 74). Propodeum golden green with blue metallic tinges (Fig. 74). Fore coxa dark brown with apical 1/3 white, mid coxa dark brown, hind coxa purple metallic (Fig. 78); femora, tibiae and tarsi on all legs white. Wings without pigmented areas; WIP in fore wing almost unicoloured, gradually changing hue from blue to green towards the margin when the membrane becomes gradually thinner (Fig. 80). Gaster with first two tergites golden green, remaining tergites golden purple with green metallic tinges.

**Figures 82–87.**
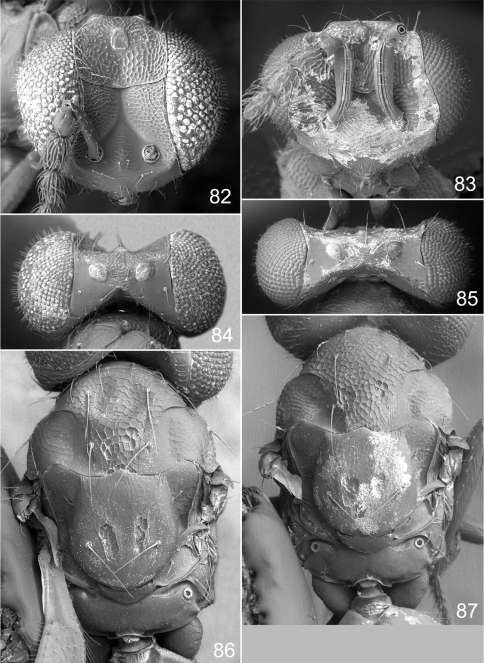
*Achrysocharoides* *serotinae* sp. n.: **82** Head frontal, female **83** Ditto, male **84** Vertex, female **85** Ditto, male **86** Mesosoma dorsal, female **87** Ditto, male.

Antenna as in Fig. 74. Frons below level of toruli smooth and shiny (Fig. 82), between level of toruli and frontal suture with raised and strong reticulation with antennal scrobes smooth, above frontal suture with raised and strong reticulation. Vertex inside ocellar triangle with engraved and weak reticulation, outside ocellar triangle smooth and shiny (Fig. 84). Occipital margin rounded.

Pronotal collar without transverse carina (Fig. 86). Mesoscutum with raised and strong reticulation (Fig. 86), meshes of reticulation smaller on sidelobes than on midlobe, midlobe with singular pits (i.e. with very strong reticulation) posteromedially; notauli as smooth impressions in posterior 2/3. Scutellum with very weak reticulation and shiny, smooth along posterior margin, with 2–4 pits medially on either side of imaginary median longitudinal line (Fig. 86). Dorsellum flat and smooth, anterolaterally with two foveae. Propodeum smooth and shiny (Fig. 86); propodeal callus with three setae. Fore wing speculum closed below. Petiole conical without shoulders.

**Ratios**. HE/MS/WM = 3.7/1.0/1.6; POL/OOL/POO = 1.7/1.0/1.0; WH/WT = 1.2; LW/LM/HW = 1.6/1.0/1.0; PM/ST = 1.0; MM/LG = 0.8–0.9.

*Male*. Length 1.4 mm. Scape and pedicel white; flagellum dark brown (Fig. 75). Frons green metallic (Fig. 73B). Vertex inside ocellar triangle golden red, outside ocellar triangle golden green. Mesoscutum golden green with anterior part blue (Fig. 77). Scutellum golden green with golden red tinges and with lateral parts blue (Fig. 77). Propodeum golden green with golden red tinges (Fig. 77). Fore coxa dark brown with apical 1/3 white, mid coxa dark brown, hind coxa purple metallic (Fig. 79); femora, tibiae and tarsi on all legs white. Wings without pigmented areas; WIP very similar to that of the female (Fig. 81). Gaster with tergites 1–2 dark brown with golden green tinges, remaining tergites dark brown with weak metallic tinges, over tergites 1–3 with a large median white spot.

Antenna as in Fig. 75, i.e. scape with about same width throughout. Frons with raised and strong reticulation, some parts with transverse striation (Fig. 83); antennal scrobes joining on frontal suture; transverse ridge evenly curved. Vertex inside ocellar triangle with engraved and very weak reticulation (Fig. 85), outside ocellar triangle smooth and shiny; anterior part with a row of 3–5 long and forward directed setae. Occipital margin rounded.

Pronotal collar without transverse carina (Fig. 87). Mesoscutum with raised and strong reticulation (Fig. 87), meshes of reticulation smaller on sidelobes than on midlobe, midlobe with pits (i.e. with very strong reticulation) posteromedially; notauli as smooth impressions in posterior 2/3. Scutellum with weak reticulation, smooth along posterior and lateral margins, with 2–5 pits medially on either side of imaginary median longitudinal line (Fig. 87). Dorsellum flat and smooth, anterolaterally with two foveae. Propodeum smooth and shiny (Fig. 87); propodeal callus with three setae. Fore wing speculum closed below. Petiole conical without shoulders.

**Ratios**. HE/MS/WM = 2.5/1.0/1.3; POL/OOL/POO = 4.6/1.8/1.0; WH/WT = 1.1; LW/LM/HW = 1.6/1.0/1.0; PM/ST = 1.0; MM/LG = 0.9.

#### Etymology.

Named after black cherry (*Prunus serotina*), the host plant.

#### Distribution.

U.S.A. (Connecticut).

#### Host.

*Phyllonorycter propinquinella* (Braun) (Lepidoptera: Gracillariidae) on black cherry (*Prunus serotina*).

##### Identification of the new species

In the most recent key to North American *Achrysocharoides* by Kamijo (1991) the two newly described species both key out to the *clypeatus* group. To include them in the key to species of this group the following changes can be made:

Females of both species run to couplet 3, alternative 2 (where *Achrysocharoides arienascapus* falls out). The second alternative is changed to lead to 3a instead of *Achrysocharoides arienascapus* and then:

**Table:** 

3a	Fore coxa and scape predominantly brown (Figs 74, 78)	*Achrysocharoides serotinae* sp. n.
–	Fore coxa and scape white	3b
3b	Entire frons above frontal suture with raised and strong reticulation (Fig. 66); scutellum with very weak and superficial reticulation (Fig. 70)	*Achrysocharoides maieri* sp. n.
–	Frons strongly reticulate medially and weakly reticulate laterally; scutellum with strong reticulation	*Achrysocharoides arienascapus* (Miller)
	Males run to couplet 4:
4	Frons above frontal suture with many short and scattered setae (see fig. 5 in Kamijo (1991)); scape with long dense setae ventrally (see fig. 5 in Kamijo (1991))	*Achrysocharoides hirtiscapus* (Miller)
–	Frons above frontal suture bare (Figs 67, 83); scape with a few short setae along ventral edge (Figs 59, 75)	5a
5a	Vertex with long setae about as long as distance between posterior ocelli (Figs 69, 85)	5b
–	Vertex with long setae at least as long as width of vertex (see fig. 7 in Kamijo (1991))	5
5b	Scape widest close to base (Fig. 59); fore coxa white (Fig. 63)	*Achrysocharoides maieri* sp. n.
–	Scape with about same width throughout (Fig. 75); fore coxa predominantly brown (Fig. 79)	*Achrysocharoides serotinae* sp. n.

## Supplementary Material

XML Treatment for Achrysocharoides maieri

XML Treatment for Achrysocharoides serotinae
